# Solvent-Free Synthesized Monolithic Ultraporous Aluminas for Highly Efficient Removal of Remazol Brilliant Blue R: Equilibrium, Kinetic, and Thermodynamic Studies

**DOI:** 10.3390/ma14113054

**Published:** 2021-06-03

**Authors:** Huan Xu, Guilhem Boeuf, Zixian Jia, Kairuo Zhu, Mehrdad Nikravech, Andrei Kanaev, Rabah Azouani, Mamadou Traore, Abdellatif Elm’selmi

**Affiliations:** 1 Laboratoire des Sciences des Procédés et des Matériaux, CNRS, Université Sorbonne Paris Nord, F-93430 Villetaneuse, France; huan.xu@lspm.cnrs.fr (H.X.); mehrdad.nikravech@lspm.cnrs.fr (M.N.); andrei.kanaev@lspm.cnrs.fr (A.K.); 2 EBInnov, École de Biologie Industrielle, F-95000 Cergy, France; g.boeuf@hubebi.com (G.B.); r.azouani@hubebi.com (R.A.); 3 CAS Key Laboratory of Photovoltaic and Energy Conservation Materials, Institute of Plasma Physics, Chinese Academy of Sciences, P.O. Box 1126, Hefei 230031, China; krzhu@mail.ustc.edu.cn

**Keywords:** ultraporous aluminas, RBBR, kinetic models, diffusion models, isotherm models, thermodynamics

## Abstract

In this study, ultraporous aluminas (UPA) were synthesized as new effective adsorbents for Remazol Brilliant Blue R (RBBR) removal from aqueous solutions. The UPA monoliths were grown via facile oxidation process, followed by isochronous annealing treatment in air at different temperatures, through which γ, θ, and α phase polycrystalline fibrous grains of UPA can be accordingly obtained. The experimental factors that affect the material adsorption performances including initial pH, contact time, and temperature were comprehensively studied by batch experiments. The RBBR adsorption isotherms of UPA(γ) and UPA(θ) powders were found almost identical, while UPA(α) powders showed low effectiveness. To obtain the desirable mechanical stability of the UPA monolith with considerable RBBR adsorption capacity, UPA(θ) powders were further studied. The UPA(θ) powders exhibited maximum RBBR adsorption at pH 2 due to the positively charged surface under acidic conditions. Compared with the Lagergren pseudo-first-order model, the pseudo-second-order model was found to explain the adsorption kinetics better. Despite the film diffusion dominating the adsorption process, the contributions of the intraparticle diffusion and chemical reactions were also found significant. The adsorption equilibrium data at different temperatures were fitted by the Langmuir, Freundlich, Temkin, and Dubinin–Radushkevich (D–R) isotherm models. The Langmuir model was found the most effective in the description of equilibrium data, and the maximum RBBR adsorption capacity retained by UPA(θ) powders was 122.55 mg·g^−1^ at 295 K. Thermodynamic parameters (ΔG^0^, ΔH^0^, and ΔS^0^) indicated the adsorption process was spontaneous and exothermic in nature.

## 1. Introduction

Throughout history, textile dyeing can be dated back to the Neolithic period (also known as the New Stone Age), followed by the serendipitous discovery of the first synthetic dye, mauveine (aniline purple) by William Perkin in 1856 [1]. Since then, the practice of employing synthetic dyes in the printing and dyeing process of fabrics has been extensively developed worldwide. At present, >7 × 10^5^ tons of dyes are produced annually, and nearly 10–15% of the total dyes are discharged in the surrounding environment with or without partial treatments, making the dyestuff-related industries responsible for up to 20% of industrial water pollution [2,3]. In addition to the textile industry, other kinds of dye-relevant industries (e.g., paper and pulp, plastic, leather, cosmetics, pharmaceutics, and photography) are also recognized as the most polluting industries. Accompanying the great conveniences brought by dye-based industrial applications, some serious problems are also caused owing to their carcinogenicity, genotoxicity, and/or mutagenicity in nature, which poses numerous threats to the ecological system and living organisms [3,4,5,6,7]. Therefore, proper treatments regarding purifications and remediations of industrial textile effluents are urgent and necessary.

The majority of dyes industrially used nowadays are organic compounds with complex and reinforced structures, which normally consist of two major components, i.e., chromophore and auxochrome groups [2,6,7]. The persistent structure and poor biodegradability of these refractory organics make the choice of an appropriate dye treatment method challenging [2]. Generally, the most known and extensively applied methods for the treatment of dyehouse effluents can be classified into physical (adsorption, membrane filtration, and reverse osmosis), chemical/electrochemical (adsorption, coagulation, flocculation, and advanced oxidation processes), biological (intracellular, and isolated enzymes) methods, and/or emerging combination of several above-mentioned techniques with the purpose of synergistic effects [4,5,6,7]. Among the available conventional methods for dye treatment, chemisorption involving chemical reactions between solutes and the functional groups on adsorbent surfaces in aqueous solutions has been confirmed and extensively studied as the most effective process for the treatment of industrial textile effluents [4,6,7].

Mesoporous materials of 2–50 nm pore diameter, owing to their numerous advantages including ordered, homogenous pore distributions, regular and tunable pore sizes, high specific surface areas, framework/wall substitutions with various metal oxides, favorable biocompatibility, and low toxicity, have been widely applied in the research field of wastewater treatment, catalyst support, drug delivery, and energy-related aspects, etc. [8,9,10]. Besides the size of pores inside the building framework, the compositions of material can vary, including pure organics (e.g., porous polymers), organic/inorganic (e.g., metal–organic frameworks, MOFs), and pure inorganics (e.g., silica, alumina, and titania). The great advantage of inorganic materials, in comparison with other materials, is that they can be synthesized relatively cheaply and usually by simple synthesis procedures [4,6,7,11,12,13]. For example, Khoshhesab et al. synthesized NiO nanoparticles by chemical precipitation method and studied its adsorption capacity for Remazol Brilliant Blue R (RBBR, also called RB19) with commercial NiO powders as a comparison [13]. Moussavi et al. synthesized porous MgO nanoparticle (nano-MgO) by sol–gel method and applied it for both azo and anthraquinone (AQ) dye removal from industrial wastewaters [7]. In particular, Madrakian et al. successfully synthesized magnetite-modified multiwalled carbon nanotubes (MMMCNTs) for the removal of four anionic dyes including RB19, Methylthymol blue, Congo red, and Mordant Blue 29 [4]. Recently, Beauvy et al. developed a new method for the synthesis of ultraporous alumina (UPA) monolith, which was followed by isochronous annealing treatment in air at different temperatures, anhydrous monolithic UPA with different crystallizations can be accordingly obtained [11,12,14,15]. The versatile applications of UPA materials with regard to photocatalytic, optical, electronic, and arsenic wastewater treatment performances have been extensively studied [16,17,18,19,20].

In the present study, RBBR was chosen as a representative AQ dye, which widely exists in industrial textile effluents. The UPA powders with different polycrystalline phases (i.e., γ, θ, and α) were synthesized for RBBR removal, and the experimental factors that affect the material adsorption performances were comprehensively studied by batch experiments. The adsorption kinetic models including the Lagergren pseudo-first-order, pseudo-second-order, film diffusion (Boyd plot), and intraparticle diffusion (Weber and Morris plot) models were applied to fit the kinetic data. Additionally, the adsorption equilibrium data at different temperatures were fitted by using the Langmuir, Freundlich, Temkin, and Dubinin–Radushkevich (D–R) isotherm models. The present study provides an improved understanding of the UPA potentials in wastewater treatment, which can explore its applications in the environmental field.

## 2. Materials and Methods

### 2.1. Chemicals and UPA Monolith Synthesis

Figure S1a,b showed the physicochemical characteristics of RBBR and its molecular compositions (wt%) including C (42.17%), H (2.57%), N (4.47%), Na (7.34%), O (28.09%), and S (15.35%) [21]. The chemicals including acetone, Hg(NO_3_)_2_, AgNO_3_, sodium acetate, CH_3_COOH, and NaOH were purchased from Sigma-Aldrich, Inc. (St. Louis, MO, USA). The raw laminated metallic aluminum plate (100 × 100 mm, 1.0 mm of thickness, 99.99% of purity) was supplied by Goodfellow Cambridge Ltd. (Huntingdon, UK). All the chemicals used in this study were of analytical grade and used as received directly without further purification. Milli-Q water (Figure S2, Millipore Corp., Burlington, MA, USA) with a specific resistivity of 18.2 MΩ·cm^−1^ at 25 °C was used to prepare solutions throughout the experiments.

The UPA monolith samples were synthesized via a facile oxidation process according to the previous studies (Figure S3) [11,12,16,17,22]. Briefly, high purity but fragile UPA monolith samples were obtained with a growth rate of ~1 cm·h^−1^ at room temperature in a humid atmosphere (70–80% RH) by the oxidation of metallic aluminum plates through a liquid layer of mercury–silver amalgam (Figure S4) [11]. Anhydrous monolithic UPA can be obtained from fragile UPA, converting to amorphous UPA, polycrystalline UPA(γ), UPA(θ), and UPA(α) monolith under 4 h of an isochronous annealing treatment in air at <870, 950, 1150, and 1350 °C, respectively (Figure S5) [11,12,17,18,22]. The mechanical stability of the UPA materials increased with the increasing calcination temperature at the expense of the specific surface area, which decreased from 300 m^2^·g^−1^ (raw fragile UPA) to 202 m^2^·g^−1^ of UPA(γ), 93 m^2^·g^−1^ of UPA(θ), and 6 m^2^·g^−1^ of UPA(α) (Figure S6). Figure S7a,b showed the adsorption isotherm profiles regarding RBBR adsorption capacity with units as mg·g^−1^ and mg·m^−2^ retained by UPA(γ), UPA(θ), and UPA(α) powders, respectively. These results showed that the RBBR adsorption capacity was proportional to the UPA specific surface area and attained approximately 1.1 mg·m^−2^ regardless of its polycrystalline phase. Therefore, to obtain the desirable mechanical stability of UPA monolith with considerable RBBR adsorption capacity, in the following studies, UPA(θ) monolith was employed to achieve more homogeneous adsorbent dispersion after a rigorous grinding process.

### 2.2. Characterization

The obtained samples were characterized by using scanning electron microscopy (SEM, Zeiss Supra 40 VP, Carl Zeiss, Jena, Germany) and transmission electron microscopy (TEM, JEOL 2011, JEOL Ltd., Tokyo, Japan) techniques. The material compositions before and after RBBR adsorption were analyzed by elemental mapping with energy-dispersive X-ray (EDX) spectroscopy (SEM S440, LEICA, Germany). The Fourier transform infrared (FTIR) spectra of the obtained samples were recorded by using a PerkinElmer Spectrum 100 system spectrometer (PerkinElmer, Waltham, MA, USA) in pressed KBr pellets (Sigma-Aldrich, St. Louis, MO, USA, 99%, analytical reagent) and in the 400–4000 cm^−1^ region. The powder X-ray diffraction (XRD) pattern of the obtained samples was carried out by using an Inel Equinox 1000 X-ray diffractometer (Inel, Celje, Slovenia) with Co Kα radiation source (λ = 1.7902 Å), and the analysis was performed at 2θ diffraction angles from 25° to 85° at a speed of 2°/min. The Brunauer–Emmett–Teller (BET) specific surface area and pore size distribution of the obtained samples were studied by nitrogen adsorption–desorption measurement (Belsorp-max, MicrotracBEL, Japan; data analysis: MicroActive for ASAP 2460) with outgassing at 200 °C for 12 h. The mass of UPA(γ), UPA(θ), and UPA(α) powders and the corresponding range of points (P/P_0_) used for the BET measurements were 0.0602 (0.0052–0.9888), 0.0586 (0.0100–0.9898), and 0.4335 g (0.0063–0.9948 pressure), respectively. The zeta potential values of the obtained samples as a function of pH were measured by a Nano ZS90 Zetasizer (Malvern Instruments Ltd., Malvern, UK). The desired pH values of suspensions between 2 and 12 were adjusted by adding negligible volumes of 0.1–0.01 mol·L^−1^ HCl (2.0–3.5), CH_3_COOH (3.5–7.0), or NaOH solution.

### 2.3. Batch Adsorption Studies

The complete experimental details can be found in the Supplementary Materials. The RBBR concentration in the supernatant (C_t_, mg·L^−1^) was determined by spectrophotometry method at the wavelength of 590 nm (Figure S1c,d, UviLine 9400 UV–Visible spectrophotometer, Secomam, France). The adsorption percentage (%), the adsorption capacity at equilibrium (*q*_e_, mg·g^−1^), and the distribution coefficient (*K*_d_) were obtained from the following equations, respectively:

(1) $$\mathrm{Adsorption}\;(\%)=\frac{C_{0}-C_{e}}{C_{0}}\times 100$$

(2) $$q_{e}=\frac{C_{0}-C_{e}}{m}\times V$$

(3) $$K_{d}=\frac{C_{0}-C_{e}}{C_{e}}\times \frac{V}{m}=\frac{q_{e}}{C_{e}}$$

where *C*_0_ (mg·L^−1^) is the initial adsorbate concentration in suspension, *C*_e_ (mg·L^−1^) is the adsorbate concentration in the supernatant at equilibrium, *V* (L) is the volume of suspension, and *m* (g) is the mass of adsorbent. All of the experimental data are the averages of triplicate determinations.

### 2.4. Data Analysis

#### 2.4.1. Adsorption Kinetic Study

The kinetic data were fitted by the Lagergren pseudo-first-order and pseudo-second-order models by using the following linearized equations, respectively [23,24]:

Lagergren pseudo-first-order model:

(4) $$\ln(Q_{m}-Q_{t})=\ln Q_{m}-k^{\prime }t$$

pseudo-second-order model:

(5) $$\frac{t}{Q_{t}}=\frac{1}{k^{\prime \prime }Q_{m}{}^{2}}+\frac{t}{Q_{m}}$$

where *Q*_t_ and *Q*_m_ (mg·g^−1^) refer to the adsorption capacity at time *t* (h) and at equilibrium obtained from the kinetic models. *k*′ (h^−1^) and *k*″ (g·mg^−1^·h^−1^) are the adsorption rate constants obtained from the kinetic models.

#### 2.4.2. Rate-Limiting Step Determination Study

In order to identify the bottleneck (slowest) step of the adsorption process, both the first-curved and second-linear adsorption parts of the kinetic data were fitted by the film diffusion (Boyd plot) and intraparticle diffusion models (Weber and Morris plot) by using the following linearized equations, respectively [25,26,27]:

Film diffusion model [25,26]:

(6) $$\ln(1-\frac{Q_{t}}{Q_{m}})=-k_{\mathrm{FD}}t$$

Intraparticle diffusion model [27]:

(7) $$Q_{t}=k_{\mathrm{IPD}}t^{0.5}+C_{\mathrm{IPD}}$$

where *Q*_t_/*Q*_m_ is the fractional attainment of equilibrium. *k*_FD_ (min^−1^) and *k*_IPD_ (mg·g^−1^·min^−0.5^) are the rate constants obtained from the diffusion models. *C*_IPD_ is proportional to the boundary layer, which provides information about boundary layer thickness, i.e., the larger value of *C*_IPD_, the greater of boundary layer effect on the adsorption process [27,28].

#### 2.4.3. Adsorption Equilibrium Study

To obtain a better understanding of the adsorption mechanisms, the adsorption equilibrium data were fitted by Langmuir, Freundlich, Temkin, and D–R isotherm models [29].

The linearized equation of the Langmuir isotherm model is listed as follows [30,31,32]:

Langmuir isotherm model:

(8) $$\frac{C_{e}}{q_{e}}=\frac{1}{K_{L}q_{e,\max}}+\frac{C_{e}}{q_{e,\max}}$$

where *q*_e,max_ (mg·g^−1^) is the maximum adsorption capacity obtained from the isotherm models, and *K*_L_ (L·mg^−1^) is the constant of the Langmuir isotherm model related to the adsorption energy. The essential characteristics of the Langmuir model can be expressed in terms of a dimensionless constant, commonly known as separation factor or equilibrium parameter (*R*_L_), which is defined by the following equation [33,34]:

(9) $$R_{L}=\frac{1}{1+K_{L}C_{0}}$$

According to Hall et al. [33], the magnitude of R_L_ indicates the adsorption process in nature to be either unfavorable (*R*_L_ > 1), linear (*R*_L_ = 1), favorable (0 < *R*_L_ < 1) or irreversible (*R*_L_ = 0).

The linearized equation of the Freundlich isotherm model is listed as follows [35]:

Freundlich isotherm model:

(10) $$\log\;q_{e}=\log\;K_{F}+\frac{1}{n}\log\;C_{e}$$

where *K*_F_ (mg^(1−1/n)^·L^1/n^·g^−1^) and 1/n are the Freundlich constants indicating the adsorption capacity and adsorption intensity, respectively. The magnitude of 1/*n* ranges between 0 and 1, indicating the surface heterogeneity, which becomes more heterogeneous as its value approaches zero [5,29,36]. Meanwhile, 1/*n* > 1 indicates a cooperative adsorption [29].

The linearized equation of the Temkin isotherm model is listed as follows [37]:

Temkin isotherm model:

(11) $$q_{e}=B\ln K_{T}+B\ln C_{e}$$

where *B* = *RT*/b_T_ (J·mol^−1^) and *K*_T_ (L·g^−1^) are the constant and equilibrium binding constant of the Temkin isotherm model, respectively.

The linearized equation of the D–R isotherm model is listed as follows [38]:

D–R isotherm model:

(12) $$\ln q_{e}=\ln q_{e,\max}-\beta \varepsilon^{2}$$

(13) $$\varepsilon =RT\;\ln(1+\frac{1}{C_{e}})$$

where *β* (mol^2^·kJ^−2^) is the constant of the D–R isotherm model, ε is the constant of the D–R isotherm model related to the Polanyi potential, *R* (8.3145 J·mol^−1^·K^−1^) is the universal gas constant, and *T* (K) is the absolute temperature in Kelvin. *E* (kJ·mol^−1^) is defined as the free energy change required to transfer 1 mol of adsorbate from infinity in solution to the solid surface [28]. This relationship can be described as follows [39]:

(14) $$E=\frac{1}{\sqrt{2\beta }}$$

The magnitude of E is useful to estimate the mechanism of the adsorption reaction. If the value of E is in the range of 8–16 kJ·mol^−1^, the adsorption process is governed by an ion-exchange mechanism, while in the case of *E* < 8 kJ·mol^−1^, the adsorption process may be affected by physical forces. On the other hand, the adsorption process may be dominated by particle diffusion if the value of *E* is greater than 16 kJ·mol^−1^ [40,41].

#### 2.4.4. Adsorption Thermodynamic Study

The thermodynamic parameters including standard Gibbs free energy (Δ*G*^0^, kJ·mol^−1^), standard enthalpy change (Δ*H*^0^, kJ·mol^−1^), and standard entropy change (Δ*S*^0^, J·mol^−1^·K^−1^) were obtained from the following equations, respectively:

(15) $$\Delta G^{0}=-RT\ln K^{0}$$

(16) $$\ln K^{0}=\frac{\Delta S^{0}}{R}-\frac{\Delta H^{0}}{RT}$$

where ln*K*^0^ (*K*^0^, the standard distribution coefficient) can be obtained by plotting ln*K*_d_ versus *C*_e_ and extrapolating *C*_e_ to zero. The slope and intercept of the plot of ln*K*^0^ versus 1000/*T* correspond to –Δ*H*^0^/1000*R* and Δ*S*^0^*/R*, respectively.

## 3. Results and Discussions

### 3.1. Characterization

**Figure 1:**
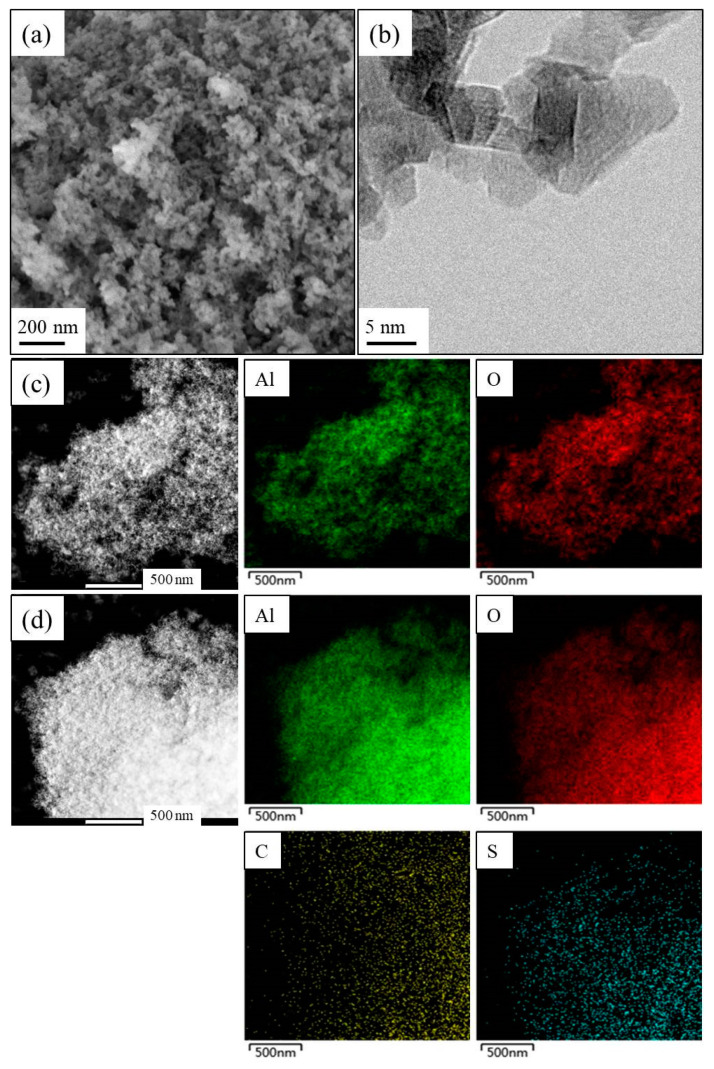
(**a**) SEM and (**b**) TEM images of UPA(θ) powders, and elemental mapping of UPA(θ) powders (**c**) before and (**d**) after RBBR adsorption.

Figure 1a,b showed the SEM and TEM images of UPA(θ) powders, which evidenced the ultraporous morphology of obtained samples. The EDX spectra of UPA(θ) powders showed that there were no residual mercury or silver element retained on the surface of obtained samples (Figure S8). As shown in Figure S9, the UPA(θ) border became smoother after RBBR adsorption, and the corresponding TEM elemental mappings in Figure 1c,d clearly confirmed the uniform distribution of RBBR retained on UPA(θ) surfaces.

**Figure 2:**
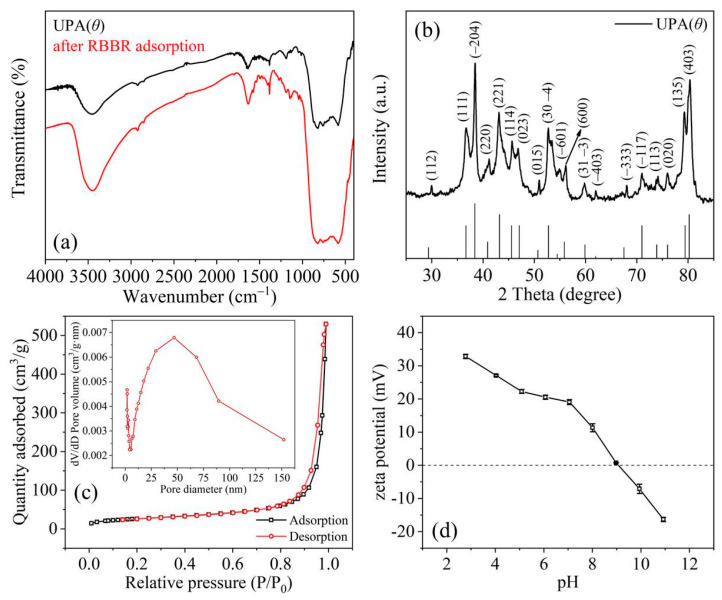
(**a**) FTIR spectra of UPA(θ) powders before and after RBBR adsorption; (**b**) XRD pattern and (**c**) nitrogen adsorption–desorption isotherm of UPA(θ) powders (inset: pore size distribution); (**d**) zeta potential value of UPA(θ) powders as a function of pH. m/V_[UPA(θ)]_ = 4.44 g·L^−1^, I = 100 mmol·L^−1^ sodium acetate.

In Figure 2a, the absorption band in the range of 3400–3500 cm^−1^ was assigned to the –OH stretching vibration of UPA(θ) powders, and the broader absorption band in this range indicated the presence of carboxyl and amino groups distributed on the UPA(θ) surface after RBBR adsorption (Figure S1a). Based on the calculation from Bragg equation (2 dsinθ = nλ, n = 1, 2, 3, etc.) (Figure 2b), the typical XRD patterns at 2θ = 36.64° (d = 0.28 nm), 38.46° (d = 0.27 nm), and 80.32° (d = 0.14 nm) correspond to the (111), (−204), and (403) planes of theta alumina, respectively, (JCPDS 35–0121, 11–0517, and 23–1009) [42]. The nitrogen adsorption–desorption isotherm curve of UPA(θ) powders followed the typical characteristics of type IV isotherm and H3 hysteresis (IUPAC), indicating the mesoporous property of UPA(θ) powders (Figure 2c) [43,44]. Moreover, the specific surface area of UPA(θ) powders was 93 m^2^·g^−1^ with an average pore diameter of 35 nm (Gaussian curve from 0–150 nm), and most data of pore size distribution located in the mesoporous range (Figure 2c). The ultraporous nature of UPA(θ) powders is expected to favor the diffusion-controlled surface reactions, which will be discussed in the following studies.

### 3.2. Initial pH Effect and Adsorption Kinetics

**Figure 3:**
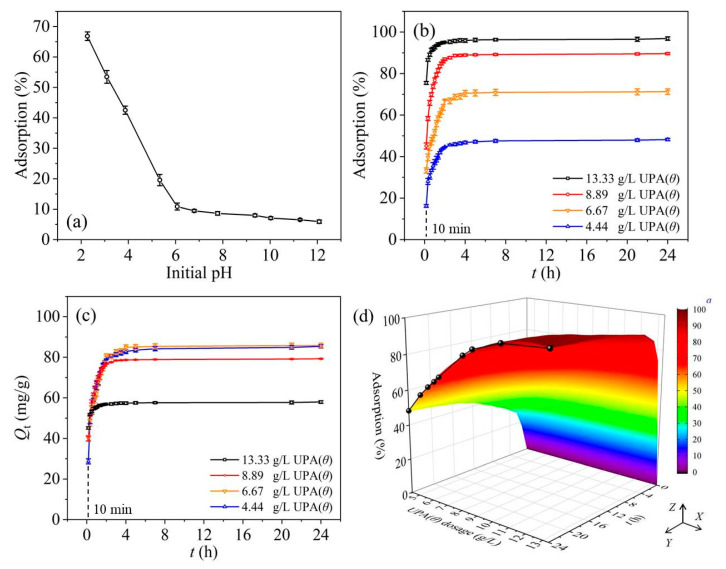
(**a**) Effect of initial pH on RBBR adsorption, C_[RBBR]initial_ = 800 mg·L^−1^, m/V_[UPA(θ)]_ = 4.44 g·L^−1^, I = 100 mmol·L^−1^ sodium acetate, T = 310 K, stirring speed = 150 rpm, and equilibrium time = 24 h. Adsorption kinetics regarding the (**b**) adsorption percentage and (**c**) adsorption capacity of RBBR retained by UPA(θ) powders; (**d**) effect of UPA(θ) dosage on RBBR adsorption kinetics. C_[RBBR]initial_ = 800 mg·L^−1^, initial pH = 4.0 ± 0.1, I = 100 mmol·L^−1^ sodium acetate, T = 310 K, stirring speed = 150 rpm, and terminal equilibrium time = 24 h. *^a^* Three-dimensional curved surface simulation based on MATLAB matrix conversion and gridding.

As shown in Figure 3a, the RBBR adsorption retained by UPA(θ) powders strongly decreased with increasing pH (up to pH = 6) and then slowly decreased in the following pH range. According to Figure 2d, the pH_zpc_ value of UPA(θ) powders approximately equaled to 9.0, and the increasing solution pH values after RBBR adsorption equilibrium indicated the significant consumption of hydrogen ions under acidic conditions (Figure S10). At low pH values, the electrostatic attraction between the protonated (positively charged) functional groups on UPA(θ) surfaces and RBBR species resulted in the high adsorption percentage of RBBR [7,45,46]. As the pH value increased, these gradually deprotonated (negatively charged) functional groups became less favorable for the adsorption process, and consequently, the affinity of UPA(θ) powders toward the negatively charged RBBR species (e.g., −SO_3_^−^, sulfonate groups) decreased (Figure 2d). Moreover, at high pH values, the formation of excessed OH^–^ ions under alkaline conditions and subsequent competition with the RBBR species for the finite reaction sites on UPA(θ) surfaces may also lead to the low RBBR adsorption percentage [7]. According to the previous studies, similar results have also been reported by applying other kinds of adsorbents for RBBR removal [6,45,46,47,48]. For example, Gök et al. found that the adsorption of RBBR onto 1,6-diamino hexane modified bentonite (DAH–bentonite) was strongly pH dependent, with the optimum pH = 1.5 [6]. Therefore, the low adsorption percentage of RBBR under alkaline conditions can be attributed to the electrostatic repulsion between the negatively charged UPA(θ) surfaces and the anionic RBBR species, which became the essential factor in controlling the adsorption process.

The kinetic data regarding the adsorption percentage (%) and adsorption capacity (Q_t_, mg·g^−1^) of RBBR retained by UPA(θ) powders were shown in Figure 3b,c, respectively. The RBBR adsorption increased rapidly in the first 4 h and then maintained a high level until the adsorption process achieved equilibrium. Figure 3b,c also showed that in the initial step, the adsorption process achieved equilibrium much more rapidly at high adsorbent dosage. In Figure 3c, the decrease of Q_t_ value of RBBR adsorption may result from the increasing UPA(θ) dosage on which more vacant reaction sites became available. In general, the RBBR adsorption process was rapid, and 4 h was enough to achieve the entire adsorption equilibrium. Based on the profiles of kinetic data (Figure 3b), the effect of UPA(θ) dosage on RBBR adsorption kinetics was shown in Figure 3d (XZ side, i.e., “UPA(θ) dosage (g·L^−1^)–Adsorption (%)” side). As discussed above, the increasing UPA(θ) dosage may result in more vacant reaction sites available on the adsorbent surfaces for RBBR adsorption. This positive relationship explained the Г line type of kinetic data on the XZ side of Figure 3d at 24 h of equilibrium time (black line highlighted). The RBBR adsorption increased with increasing UPA(θ) dosage and exceeded 96% when UPA(θ) dosage attained more than 10 g·L^−1^. As long as sufficient reaction sites were provided, the RBBR adsorption was independent of UPA(θ) dosage. All the above discussions indicated that the UPA(θ) dosage played an important role in the adsorption process; however, the economic issues usually should be taken into consideration in the actual adsorbent applications. Therefore, in order to obtain suitable dye treatment, one should determine the appropriate UPA(θ) dosage according to the initial concentration of dye effluents [6,7,49,50].

### 3.3. Adsorption Kinetic and Rate-Limiting Step Determination Studies

**Figure 4:**
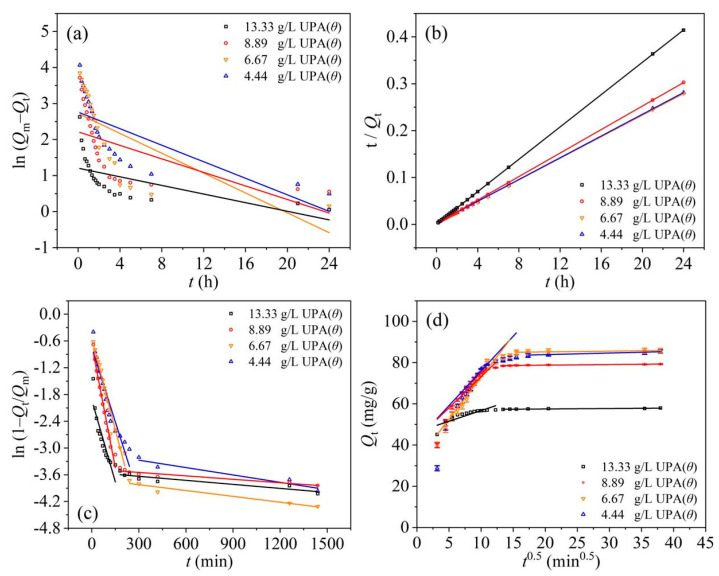
Tests of the (**a**) Lagergren pseudo-first-order, (**b**) pseudo-second-order, (**c**) film diffusion (Boyd plot), and (**d**) intraparticle diffusion (Weber and Morris plot) models on RBBR adsorption retained by UPA(θ) powders.

The fitting results of the Lagergren pseudo-first-order and pseudo-second-order models were shown in Figure 4a,b, respectively, and the fitting parameters were listed in Table 1.

As shown in Table 1, the low determination coefficients (*R*^2^) obtained from the Lagergren pseudo-first-order model (Equation (4)) showed that between the kinetic data and this model, there was no significant correlation. On the other hand, the pseudo-second-order model (Equation (5)) fitted the kinetic data better, and the calculated adsorption capacities at equilibrium (*Q*_mc_) were closer to the experimental ones (*Q*_me_). Therefore, the adsorption process followed the pseudo-second-order model based on the assumption that the rate-limiting step may be chemical adsorption or chemisorption involving valence forces through sharing or exchanging electrons between the adsorbent and adsorbate, which provides the best correlation of the kinetic data [24,51].

Generally, the adsorption kinetics of solutes retained by porous materials is controlled by different steps [50,51]: (i) solutes transfer from the aqueous phase to the more external adsorbent surface, crossing the boundary film bordering the solid adsorbent particles (film diffusion step), (ii) internal diffusion of solutes transferring from the adsorbent surface to the intraparticle active sites (particle diffusion step), and (iii) sequestration on the active sites via adsorption, complexation, or intraparticle precipitation phenomena. One or more of the above-mentioned steps may affect the mechanisms governing the adsorption process. The fitting results of the film diffusion and intraparticle diffusion models were shown in Figure 4c,d, respectively, and the fitting parameters were listed in Table 2.

The nonlinear distribution of points with two distinct regions observed in Figure 4d indicated that intraparticle/pore diffusion may participate in the adsorption process but was not the single rate-limiting step [51]. Furthermore, the deviation of intercepts of Weber and Morris plot (*C*_IPD_, Figure 4d) may be due to the difference in the rate of mass transfer in the initial and final stages of the adsorption process [28,52]. For the RBBR adsorption retained by UPA(θ) powders, the initial curved region of the plot was attributed to the film diffusion, and the subsequent linear region was attributed to the intraparticle diffusion and chemical reactions [48,51].

### 3.4. Adsorption Equilibrium Study

**Figure 5:**
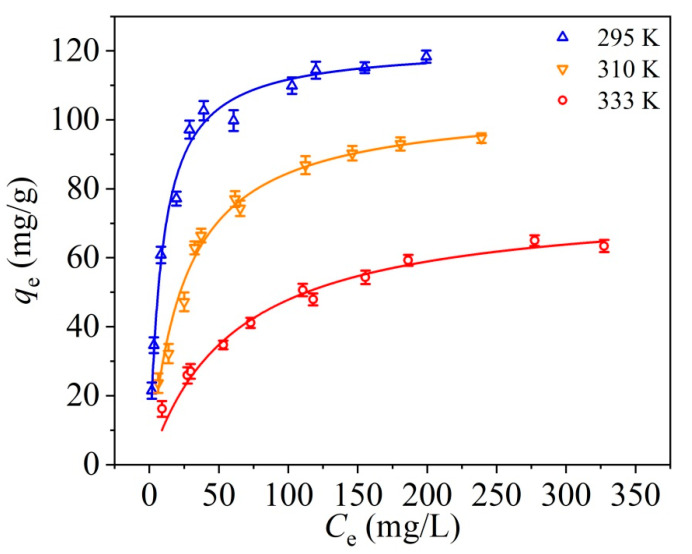
Adsorption isotherm profiles of RBBR retained by UPA(θ) powders at different temperatures. m/V_[UPA(θ)]_ = 5.56 g·L^−1^, initial pH = 4.0 ± 0.1, I = 100 mmol·L^−1^ sodium acetate, stirring speed = 150 rpm, and equilibrium time = 24 h.

As shown in Figure 5, the RBBR adsorption capacity of UPA(θ) powders decreased with increasing temperature, which indicated that the adsorption reaction may be exothermic, and low temperature favored the adsorption process.

Based on the assumption that the monolayer adsorption can only occur at a finite number of definite localized sites with no lateral interaction and steric hindrance between the adsorbed molecules, even on adjacent sites, the Langmuir model is widely used for the fitting of the homogeneous adsorption [30,31,32]. This empirical model is graphically characterized by a plateau and an equilibrium saturation point where once a molecule occupies a site, no further adsorption can take place [29]. Unlike the Langmuir model, the Freundlich model is an empirical model widely applied in heterogeneous systems (e.g., organic compounds, highly interactive species on activated carbon and molecular sieves), which describes the nonideal and reversible adsorption with no restrictions to the formation of monolayer [35]. The Temkin model, which contains a factor that explicitly takes account of the adsorbent–adsorbate interactions, was firstly introduced describing the adsorption of hydrogen onto platinum electrodes in acidic solutions [37]. By ignoring the extremely low and large value of concentrations, this model assumes that the heat of adsorption (function of temperature) of all the molecules in the layer would decrease linearly rather than logarithmically with coverage [29,53]. Compared with its less applicability to the more complex adsorption systems, especially the liquid phase adsorption isotherms, the Temkin model can be well applied for predicting the gas phase equilibrium. The D–R model is an empirical model generally applied to express the adsorption mechanism with a Gaussian energy distribution onto a heterogeneous surface [38]. This model has often successfully fitted high solute activities and the intermediate range of concentrations data well.

**Figure 6:**
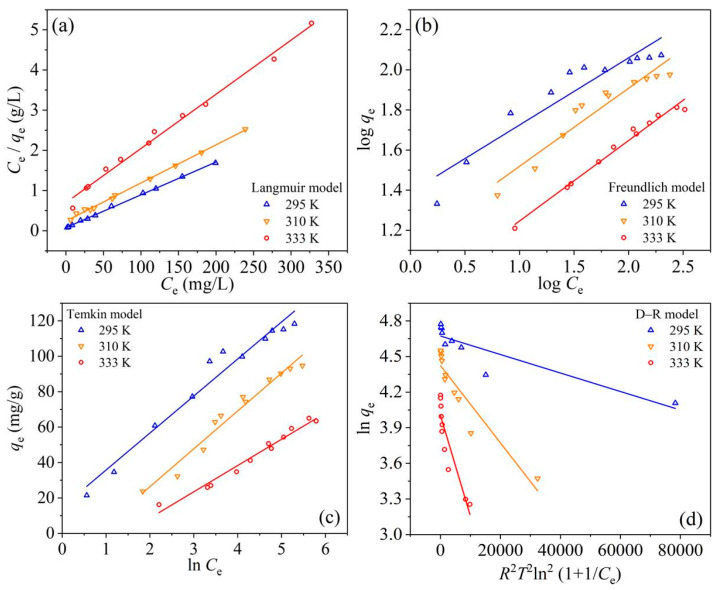
Tests of the (**a**) Langmuir, (**b**) Freundlich, (**c**) Temkin, and (**d**) D–R isotherm models on RBBR adsorption retained by UPA(θ) powders. at different temperatures.

The fitting results of the Langmuir, Freundlich, Temkin, and D–R isotherm models were shown in Figure 6, and the fitting parameters were listed in Table 3.

Compared the obtained *R*^2^ values from different isotherm models with each other, the Langmuir model fitted the adsorption equilibrium data better than the other three models (i.e., Langmuir > Temkin ≈ Freundlich > D–R model), indicating the presence of RBBR monolayer coverage on UPA(θ) surfaces [30,31,32]. Based on the Langmuir model, the *q*_e,max_ values of RBBR adsorption retained by UPA(θ) powders were 122.55, 105.49, and 74.18 mg·g^−1^ for 295, 310, and 333 K, respectively. These results were in good accordance with the equilibrium *q*_e_ values obtained from the adsorption equilibrium study (Figure 5), which was consistent with the low-temperature favorable conclusion, as discussed above. The R_L_ values listed in Table 3 fell in the range of 0–1, indicating that the RBBR adsorption retained by UPA(θ) powders was favorable, and RBBR tended to remain the bonding on UPA(θ) surfaces [33]. The obtained R^2^ values from the Temkin model were slightly greater than those of the Freundlich model, and the equilibrium binding constants (*K*_T_) at high temperatures were less than that at room temperature. In the Freundlich model, all the values of 1/*n* at different temperatures were less than 1, indicating the surface heterogeneity of UPA(θ) powders during the adsorption process [5,29,36]. Among the isotherm models applied in this study, the obtained R^2^ values from the D–R model were the lowest among the considered isotherm models, and the *q*_e,max_ values were much less than the equilibrium *q*_e_ values obtained from the adsorption isotherms (Figure 5). Consequently, the model analysis indicated the low applicability of the D–R model on the adsorption process. Therefore, the Langmuir isotherm model was found to describe the adsorption equilibrium data best, and the maximum RBBR adsorption capacity retained by UPA(θ) powders was 122.55 mg·g^−1^ at 295 K.

### 3.5. Adsorption Thermodynamic Study

**Figure 7:**
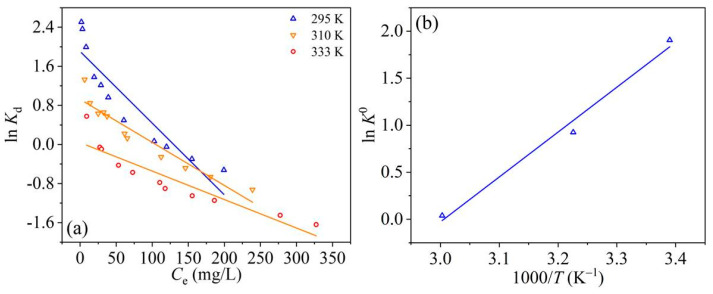
Linear plots of (**a**) ln*K_d_* versus C_e_ and (**b**) ln *K*^0^ versus 1000/T for RBBR adsorption retained by UPA(θ) powders.

The thermodynamic parameters can define whether the RBBR adsorption retained by UPA(θ) powders was endothermic or exothermic, spontaneous or not [54,55]. The linear plots of ln*K*_d_ versus C_e_ and lnK^0^ versus 1000/T were shown in Figure 7a,b, respectively. The obtained thermodynamic parameters were listed in Table 4.

In general, the positive Δ*G*^0^ values at all temperatures indicate that the adsorption process requires energy from an external source to convert reactants into products, which is considered thermodynamically unfavorable. In this study, the obtained negative Δ*G*^0^ values indicated that the RBBR adsorption process was thermodynamically favorable and spontaneous. The increase of Δ*G*^0^ value with increasing temperature indicated that low temperature favored the adsorption process. The negative Δ*H*^0^ value confirmed the exothermicity of the adsorption process. Moreover, the magnitude order of ΔH^0^ value can indicate the type of adsorption process to be either physical (2.1–20.9 kJ·mol^−1^) or chemisorption (80–200 kJ·mol^−1^) [56]. Consequently, the RBBR adsorption retained by UPA(θ) powders can be attributed to a combined physic–chemical adsorption in nature. The magnitude of Δ*S*^0^ value can be used to describe the randomness at the solid–liquid interface during the adsorption process. According to the previous studies [54,55,56], the negative value of Δ*S*^0^ reflected that the adsorption process involves an associative mechanism, and no significant change occurred in the internal structures of the adsorbent during the adsorption process, while the positive Δ*S*^0^ value reflected the affinity of the adsorbent to adsorbate species involving the dissociative mechanism. For the RBBR adsorption retained by UPA(θ) powders, the negative value of Δ*S*^0^ indicated that the adsorption process was enthalpy driven, accompanying a decreased disorder that occurred at the solid–liquid interface.

### 3.6. Adsorption Mechanism

**Figure 8:**
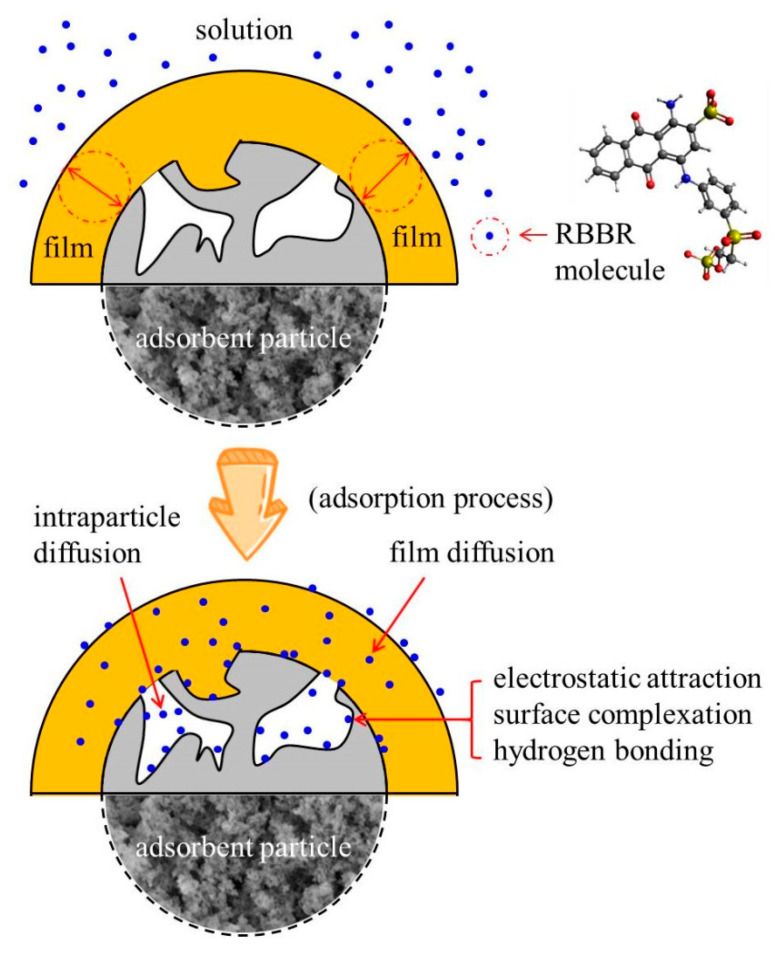
Possible mechanisms for RBBR adsorption retained by UPA(θ) powders.

Figure 8 showed the possible mechanisms for the adsorption of RBBR retained by UPA(θ) powders. Film diffusion, intraparticle diffusion, electrostatic attraction, surface complexation, and hydrogen bonding could be considered as the major interactions in the adsorption mechanisms for the removal of RBBR retained by UPA(θ) powders. The ultraporous nature of the UPA(θ) powders induced the adsorption of RBBR molecules by film and intraparticle diffusion mechanisms. The hydroxyl functional groups distributed on the UPA(θ) surface tended to form complexes with RBBR molecules by several mechanisms including electrostatic interaction and surface complexation, especially under acidic conditions. Moreover, hydrogen bonding between the hydroxyl functional groups (hydrogen bond donors) and nitrogen and/or oxygen centers in RBBR molecules (hydrogen bond acceptors) may also have some influences on the adsorption process.

Compared with the other organic, inorganic, or bio-based adsorbents reported in related studies in the literature (Table 5), UPA(θ) and UPA(γ) powders possess several advantages for large-scale applications including nontoxicity, facile synthesis, and higher RBBR adsorption capacity, compared with referenced adsorbents [4,5,6,7,13,45,46,47,49,50,57,58,59,60,61]. Therefore, UPA materials have high potentials as alternative adsorbents for the practical treatment of dye effluents.

## 4. Conclusions

In this study, UPA materials were synthesized as new effective and low-cost adsorbents for RBBR removal from aqueous solutions. The synthesized materials were characterized using FTIR, XRD, SEM, TEM, and BET. The adsorption process was pH- and temperature dependent, and the maximum RBBR adsorption capacity retained by UPA(θ) powders was 122.55 mg·g^−1^ at 295 K. Both the film diffusion and intraparticle diffusion contributed to the adsorption kinetics, and chemical reactions also played a significant role during the entire adsorption process. According to the obtained fitting results, the pseudo-second-order model and the Langmuir isotherm model were found to best describe the experimental data (i.e., pseudo-second-order > Lagergren pseudo-first-order model; and Langmuir > Temkin ≈ Freundlich > D–R isotherm model). Moreover, the thermodynamic parameters indicated that the adsorption process was spontaneous and exothermic in nature. The findings of this study highlight the UPA potentials in wastewater treatment, which can broaden our understanding and its applications in the environmental field.

## Figures and Tables

**Table 1:** Parameters of RBBR adsorption kinetics fitted by the Lagergren pseudo-first-order and pseudo-second-order models at T = 310 K.

	UPA(θ) Dosage (g·L^−1^)			
Kinetic Models	4.44	6.67	8.89	13.33
**Lagergren pseudo-first-order**				
*k*′ (h^−1^)	0.115	0.138	0.094	0.060
*Q*_mc_ (mg·g^−1^) ^a^	15.844	15.243	9.171	3.337
*R* ^2^	0.521	0.511	0.336	0.332
**Pseudo-second-order**				
*k*″ (g·mg^−1^·h^−1^)	0.049	0.046	0.102	0.422
*Q*_mc_ (mg·g^−1^) ^a^	86.207	86.957	79.745	57.803
*R* ^2^	0.999	0.999	0.999	0.999
*Q*_me_ (mg·g^−1^) ^b^	87.385	87.934	81.116	59.987

^a^*Q*_mc_ (mg·g^−1^) is the calculated adsorption capacity at equilibrium obtained from the kinetic models. ^b^ *Q*_me_ (mg·g^−1^) is the experimental adsorption capacity at equilibrium.

**Table 2:** Parameters of RBBR adsorption kinetics fitted by the film diffusion (Boyd plot) and intraparticle diffusion (Weber and Morris plot) models at T = 310 K.

	First-Curved Adsorption Part				Second-Linear Adsorption Part			
Diffusion Models	4.44	6.67	8.89	13.33	4.44	6.67	8.89	13.33
Film diffusion								
*k*_FD_ (min^−1^)	0.0111	0.0136	0.0184	0.0123	0.0006	0.0004	0.0003	0.0003
Intercept on *Y* axis ^a^	−0.746	−0.556	−0.690	−1.922	−3.108	−3.691	−3.471	−3.545
*R* ^2^	0.911	0.956	0.976	0.807	0.885	0.909	0.866	0.826
Intraparticle diffusion								
*k*_IPD_ (mg·g^−1^·min^−0.5^)	3.3628	4.0538	3.0117	1.0385	0.0769	0.0374	0.0281	0.0217
*C*_IPD_	42.309	32.299	43.003	46.347	82.332	84.429	78.199	57.072
*R* ^2^	0.708	0.904	0.873	0.704	0.899	0.916	0.875	0.838

^a^ A linear plot of −ln(1 − *Q*_t_/*Q*_m_) versus t with zero intercept indicates that the kinetics of the adsorption process is controlled by diffusion through the liquid surrounding the solid adsorbent particles.

**Table 3:** Parameters of RBBR adsorption isotherms fitted by the Langmuir, Freundlich, Temkin, and D–R isotherm models at different temperatures.

Isotherm Models	295 K	310 K	333 K
Langmuir			
q_e,max_ (mg·g^−1^)	122.549	105.485	74.184
*K*_L_ (L·mg^−1^)	0.107	0.039	0.019
*R* ^2^	0.999	0.998	0.991
*R*_L_	0.011–0.078	0.030–0.188	0.056–0.275
Freundlich			
*K*_F_ (mg^(1−1/n)^·L^1/n^·g^−1^)	24.548	13.461	6.991
1/*n*	0.335	0.390	0.401
*R* ^2^	0.880	0.903	0.982
Temkin			
*B* (J·mol^−1^)	20.892	21.376	14.824
*K*_T_ (L·g^−1^)	2.036	0.464	0.242
*R* ^2^	0.960	0.961	0.973
D–R			
*q*_e,max_ (mg·g^−1^)	107.037	83.502	54.976
*β* (mol^2^·kJ^−2^)	7.795 × 10^−6^	3.257 × 10^−5^	8.496 × 10^−5^
*R* ^2^	0.784	0.866	0.823

**Table 4:** Thermodynamic parameters of RBBR adsorption retained by UPA(θ) powders.

*T* (K)	Δ*G*^0^ (KJ·Mol^−1^)	Δ*H*^0^ (kJ·Mol^−1^)	Δ*S*^0^ (J·Mol^−1^·K^−1^)
295	−4.673		
310	−2.383	−39.711	−119.372
333	−0.107		

**Table 5:** Comparison of RBBR adsorption capacity retained by UPA materials with other organic, inorganic, or bio-based adsorbents reported in related studies in the literature.

Adsorbents	Experimental Conditions ^a^	*q*_e,max_ (Mg·g^−1^) ^b^	Ref.
Mazandaran wood waste (WW)	pH = 1.72, T = ND	4.75	[57]
ZnO nanoparticles (ZnO NPs)	pH = 3.0, T = 298 K	38.02	[49]
Commercial NiO	pH = ND, T = 298 K	38.62	[13]
NiO nanoparticles	pH = ND, T = 298 K	98.83	[13]
Magnetite/GO (MGO) nanocomposite	pH = 3.0, T = 298 K	62.50	[58]
Magnetite nanoparticles (MNPs)	pH = ND, T = 298 K	74.40	[50]
Free fungal biomass (FFB)	pH = 2.0, T = 303 K	80.91	[45]
Loofa sponge-immobilized fungal biomass (LSIFB)	pH = 2.0, T = 303 K	98.90	[45]
Magnetite-modified MWCNTs (MMMCNTs)	pH = 4.0, T = 298 K	88.80	[4]
Rhizopus arrhizus biomass	pH = 2.0, T = 298 K	90.00	[46]
Carboxylated MWCNTs	pH = ND, T = 298 K	95.24	[59]
Wheat bran	pH = 1.5, T = 293 K	97.10	[5]
Magnetite nanoparticles-modified AC (MMAC)	pH = 4.0, T = 298 K	104.60	[60]
Polypyrrole-coated Fe_3_O_4_ (Ppy@Fe_3_O_4_ MNPs)	pH = 3.0, T = 298 K	112.36	[47]
Ultraporous alumina(γ) (UPA(α))	pH = 4.0, T = 295 K	17.42	This study
Ultraporous alumina(θ) (UPA(θ))	pH = 4.0, T = 295 K	122.55	This study
Ultraporous alumina(α) (UPA(γ))	pH = 4.0, T = 295 K	212.31	This study
Modified polyethyleneimine (LMW–PEI)	pH = 10.0, T = 298 K	121.00	[61]
Modified bentonite (DAH–bentonite)	pH = 1.5, T = 293 K	134.71	[6]
MgO nanoparticles (Nano-MgO)	pH = 8.0, T = 298 K	166.70	[7]

^a^ ND: No data. ^b^ Adsorption capacity uniformly converted into mg·g^−1^ (typical unit).

## Data Availability

The data presented in this study are available from the corresponding authors with reasonable requests.

## Supplementary Materials

The following are available online at https://www.mdpi.com/article/10.3390/ma14113054/s1, Table S1: Nomenclature, Greek symbols, and subscripts, Figure S1: Physicochemical characteristics, molecular compositions, 3-D model and calibration curve of RBBR, Figure S2: Photo of the Milli-Q water system, Figure S3: Photos of raw aluminum grid, UPA monolith sample after different time of continuous growth process, Figure S4: Photo of the experimental instrument for the synthesis of UPA monolith, Figure S5: Photos of raw fragile UPA, under 4 h of isochronous annealing treatment in air at different temperatures, Figure S6: Nitrogen adsorption–desorption isotherms and pore size distributions of UPA(γ), UPA(θ), and UPA(α) powders, Figure S7: Adsorption isotherm profiles regarding RBBR adsorption capacity with units as mg·g−1 and mg·m−2, Figure S8: EDX spectra of UPA(θ) powders before and after RBBR adsorption, Figure S9: SEM images of UPA(θ) powders before and after RBBR adsorption, Figure S10: The pH variation after RBBR adsorption equilibrium, Figure S11: Zeta potential values of UPA(γ), UPA(θ), and UPA(α) powders as a function of pH, Figure S12: Infrared spectrum of RBBR.
