# Supplementary material for: Solvent-Free Synthesized Monolithic Ultraporous Aluminas for Highly Efficient Removal of Remazol Brilliant Blue R: Equilibrium, Kinetic, and Thermodynamic Studies
Source: Materials (Basel). 2021 Jun 3;14(11):3054. doi: 10.3390/ma14113054 (PMC8199940; doi:10.3390/ma14113054)
Supplement: Supplementary file 1 [file materials-14-03054-s001.zip › materials-1210270-supplementary.pdf]

Supplementary Materials

Solvent-Free Synthesized Monolithic Ultraporous Aluminas for Highly Efficient Removal of Remazol Brilliant Blue R: Equilibrium, Kinetic, and Thermodynamic Studies

Huan Xu [1,2], Guilhem Boeuf [2], Zixian Jia [1,*], Kairuo Zhu [3], Mehrdad Nikravech [1], Andrei Kanaev [1], Rabah Azouani [2], Mamadou Traore [1,*], and Abdellatif Elm'selmi [2,*]

1 Laboratoire des Sciences des Procédés et des Matériaux, CNRS, Université Sorbonne Paris Nord, F-93430 Villetaneuse, France; huan.xu@lspm.cnrs.fr (H.X.); mehrdad.nikravech@lspm.cnrs.fr (M.N.); andrei.kanaev@lspm.cnrs.fr (A.K.)

2 EBInnov, École de Biologie Industrielle, F-95000 Cergy, France; g.boeuf@hubebi.com (G.B.); r.azouani@hubebi.com (R.A.)

3 CAS Key Laboratory of Photovoltaic and Energy Conservation Materials, Institute of Plasma Physics, Chinese Academy of Sciences, P.O. Box 1126, Hefei 230031, China; krzhu@mail.ustc.edu.cn

* Correspondence: zixian.jia@gmail.com (Z.J.); mamadou.traore@lspm.cnrs.fr (M.T.); a.elmselmi@hubebi.com (A.E.); Tel.: +33-01-4940-4052 (M.T.); +33-01-8576-6716 (A.E.)

Batch Adsorption Studies

Except for the study of adsorption isotherms, the adsorption process of RBBR retained by UPA(θ) powders was carried out by batch experiments at T = 310 K. In order to achieve homogeneous dispersion, a certain amount of UPA(θ) powders was taken immediately to prepare suspension and shaken for 1 min on the multifunctional vortex oscillator before the corresponding experiment. Appropriate volumes of RBBR (10 $g{\cdot}L^{-1}$) and sodium acetate (400 $mmol{\cdot}L^{-1}$) stock solutions were added into the Eppendorf tube to achieve the desired concentrations of different components. After adding the above components and controlling the final volume of suspension in the Eppendorf tube, the initial concentrations of RBBR and sodium acetate were 800 $mg{\cdot}L^{-1}$ and 100 $mmol{\cdot}L^{-1}$, respectively. The desired pH values of suspensions between 2 and 12 were adjusted by adding negligible volumes of 0.1–0.01 $mol{\cdot}L^{-1}$ HCl (2.0–3.5), CH_3COOH (3.5–7.0), or NaOH solution. For the study of adsorption isotherms at T = 295, 310, and 333 K, the suspensions were placed in the Infors HT Minitron Incubator Shaker at determined temperature for continuous shaking. After the suspensions were shaken for a certain time interval, the solid and liquid phases were separated by centrifugation at 8000 rpm for 5 min.

Table S1. Nomenclature, Greek symbols, and subscripts.

**Nomenclature**	
%	the adsorption percentage (dimensionless)
B	the constant of the Temkin isotherm model ($J\cdot mol^{-1}$)
C_0	the initial adsorbate concentration in suspension ($mg\cdot L^{-1}$)
C_e	the adsorbate concentration in the supernatant at equilibrium ($mg\cdot L^{-1}$)
C_{IPD}	the constant proportional to the thickness of the boundary layer (dimensionless)
E	the free energy change ($kJ\cdot mol^{-1}$)
k'	the adsorption rate constant of the Lagergren pseudo-first-order model (h^{-1})
k''	the adsorption rate constant of the pseudo-second-order model ($g\cdot mg^{-1}\cdot h^{-1}$)
k_{FD}	the rate constant of the film diffusion model (min^{-1})
k_{IPD}	the rate constant of the intraparticle diffusion model ($mg\cdot g^{-1}\cdot min^{-0.5}$)
K^0	the standard distribution coefficient (dimensionless)
K_d	the distribution coefficient (dimensionless)
K_F	the constant of the Freundlich isotherm model indicating the adsorption capacity ($mg^{(1-1/n)}\cdot L^{1/n}\cdot g^{-1}$)
K_L	the constant of the Langmuir isotherm model related to the adsorption energy ($L\cdot mg^{-1}$)
K_T	the equilibrium binding constant of the Temkin isotherm model ($L\cdot g^{-1}$)
m	the mass of adsorbent (g)
1/n	the constant of the Freundlich isotherm model indicating the adsorption intensity (dimensionless)
pH_{zpc}	the zero point of charge (dimensionless)
q_e	the adsorption capacity at equilibrium ($mg\cdot g^{-1}$, or $mg\cdot m^{-2}$)
$q_{e,\ max}$	the maximum adsorption capacity obtained from the isotherm models ($mg\cdot g^{-1}$)
Q_m and Q_{mc}	the adsorption capacity at equilibrium obtained from the kinetic models ($mg\cdot g^{-1}$)
Q_{me}	the experimental adsorption capacity at equilibrium ($mg\cdot g^{-1}$)
Q_t	the adsorption capacity ($mg\cdot g^{-1}$) at time t (h, or min)
R	the universal gas constant (8.3145 $J\cdot mol^{-1}\cdot K^{-1}$)
R^2	the coefficient of determination (COD, dimensionless)
R_L	the separation factor (dimensionless)
t	the contact time (h, or min)
T	the absolute temperature in Kelvin (K)
V	the volume of suspension (L)
ΔG^0	the standard Gibbs free energy ($kJ\cdot mol^{-1}$)
ΔH^0	the standard enthalpy change ($kJ\cdot mol^{-1}$)
ΔS^0	the standard entropy change ($J\cdot mol^{-1}\cdot K^{-1}$)
Greek symbols	
α	UPA crystalline phase (1350 °C, and 4 h of isochronous annealing treatment in air)
θ	UPA crystalline phase (1150 °C, and 4 h of isochronous annealing treatment in air)
γ	UPA crystalline phase (950 °C, and 4 h of isochronous annealing treatment in air)

β	the constant of the D.-R. isotherm model ($mol^2 \cdot kJ^{-2}$)
ε	the constant of the D.-R. isotherm model related to the Polanyi potential (dimensionless)
Subscripts	
0	initial state (i.e., C_0)
e	at equilibrium (i.e., C_e, q_e, $q_{e, max}$)

2

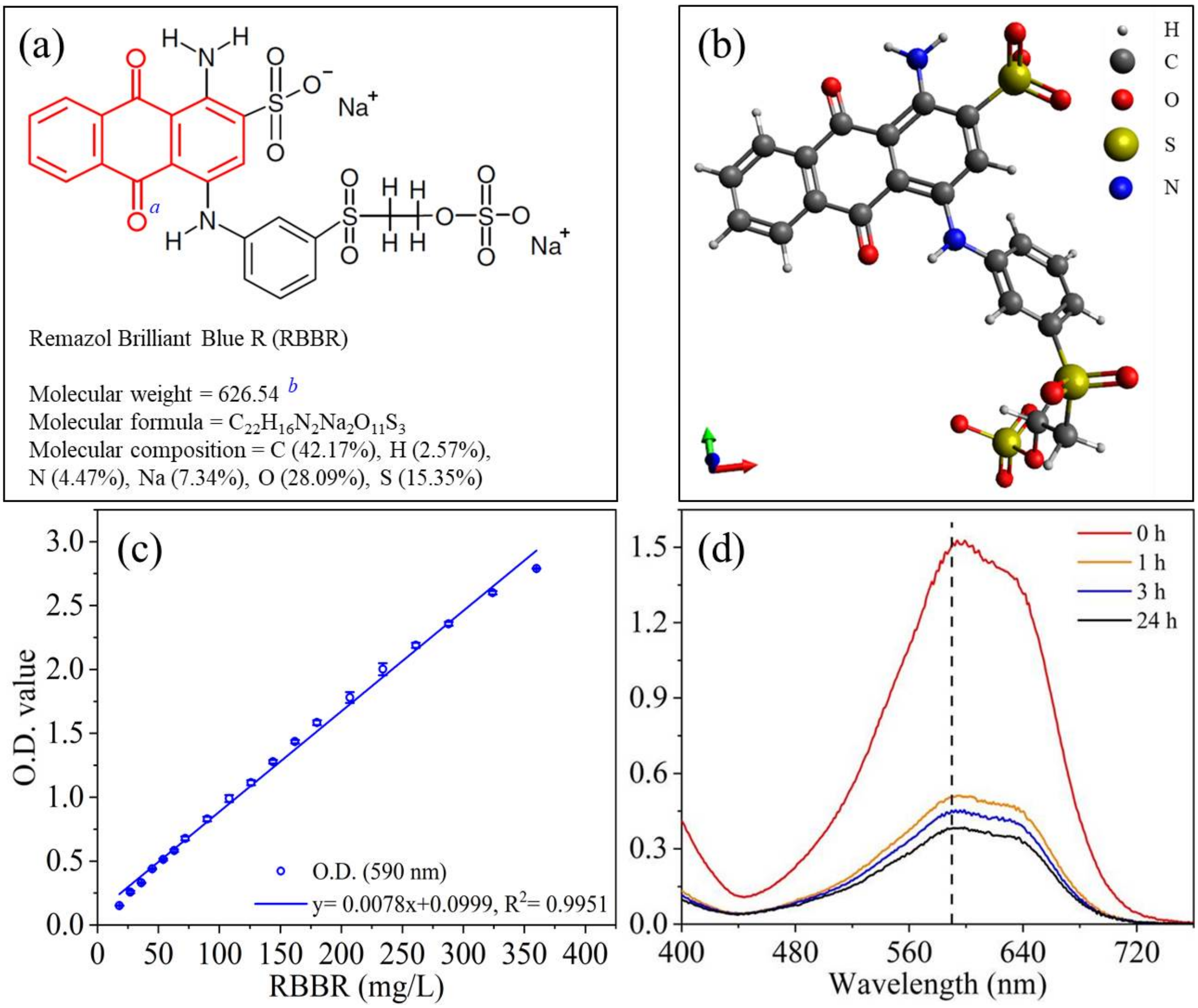

Figure S1. (**a**) Chemical structural diagram of RBBR, (**b**) 3-D model of RBBR molecule with two negative charges, (**c**) RBBR calibration curve at the wavelength of 590 nm, and (**d**) visible spectral curves of RBBR before and after adsorption retained by UPA(θ) powders monitoring with time.[a] Highlighted: Chemical structure of the anthracene-based anthraquinone group, which consists of three fused benzene rings with two carbonyl groups on the central ring. b Molecular weight in nonionized form.

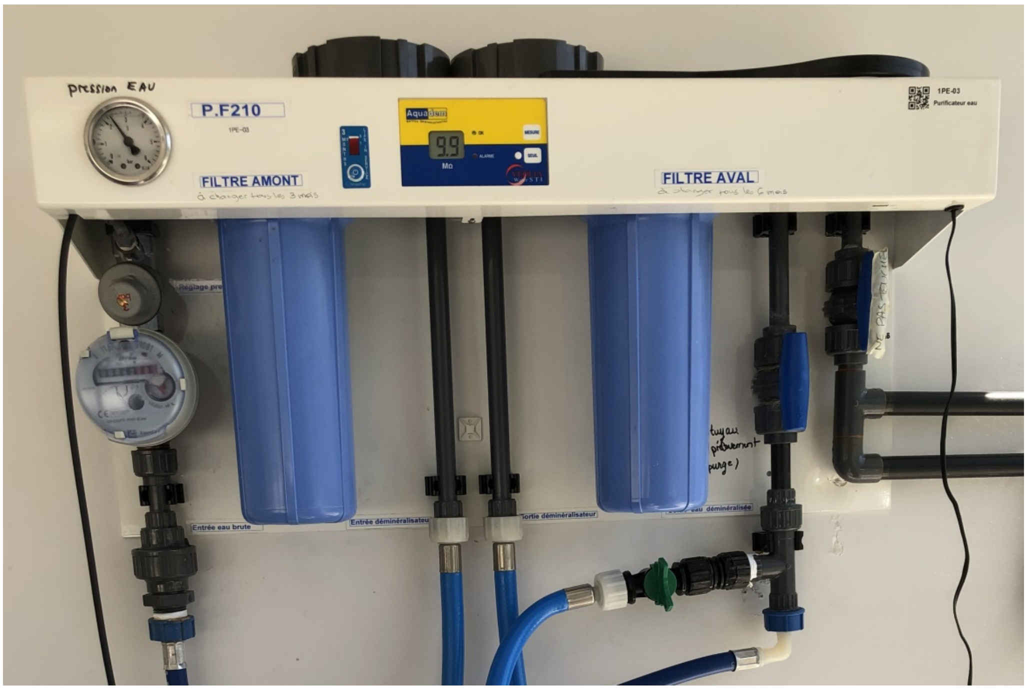

Figure S2. Photo of the Milli-Q water system (Millipore Corp.).

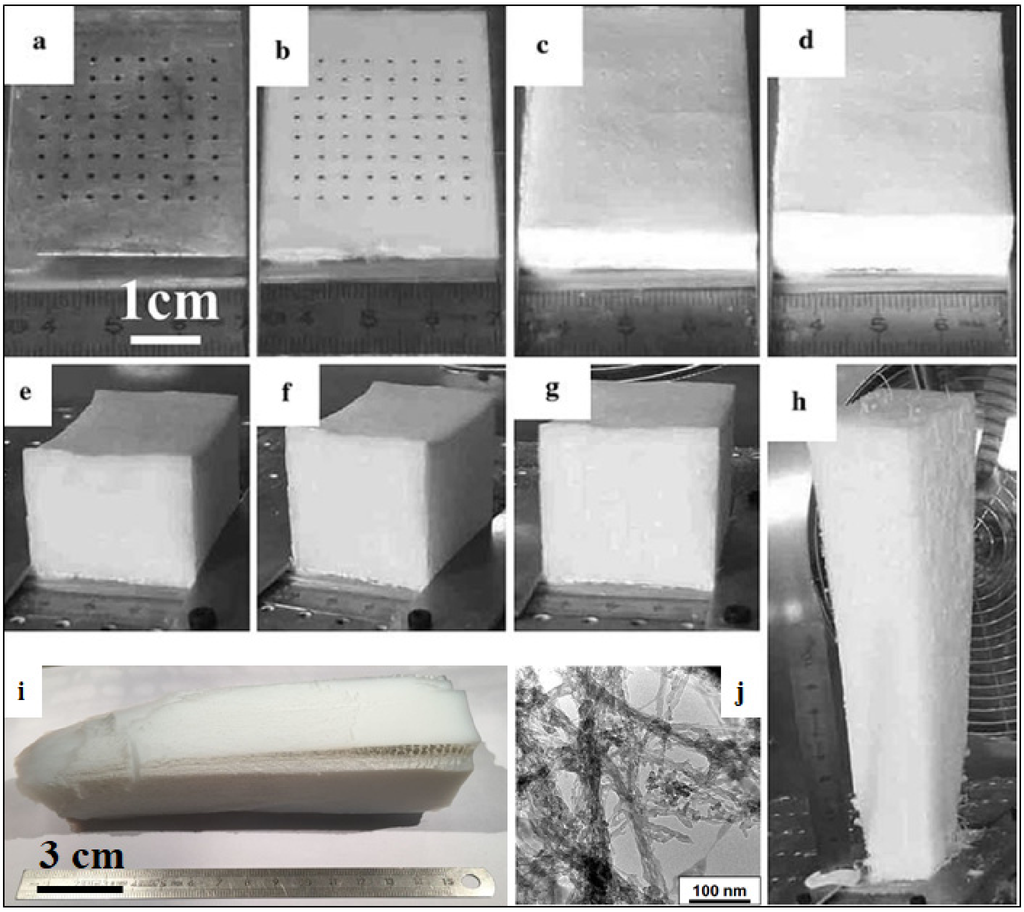

Figure S3. Photos of (**a**) raw aluminum grid, UPA monolith sample after (**b**) 10 min, (**c**) 1 h, (**d**) 2 h, (**e**) 4 h, (**f**) 5 h, (**g**) 6 h, and (**h**) 16 h of continuous growth process. T = 20 °C, RH (relative humidity) = 70%, C[$Hg(NO_3)_2$] = 0.05 mol·L^{-1}, C[$AgNO_3$] = 0.01 mol·L^{-1} [1,2]. (i) Photo and (**j**) TEM images of UPA monolith sample [3].

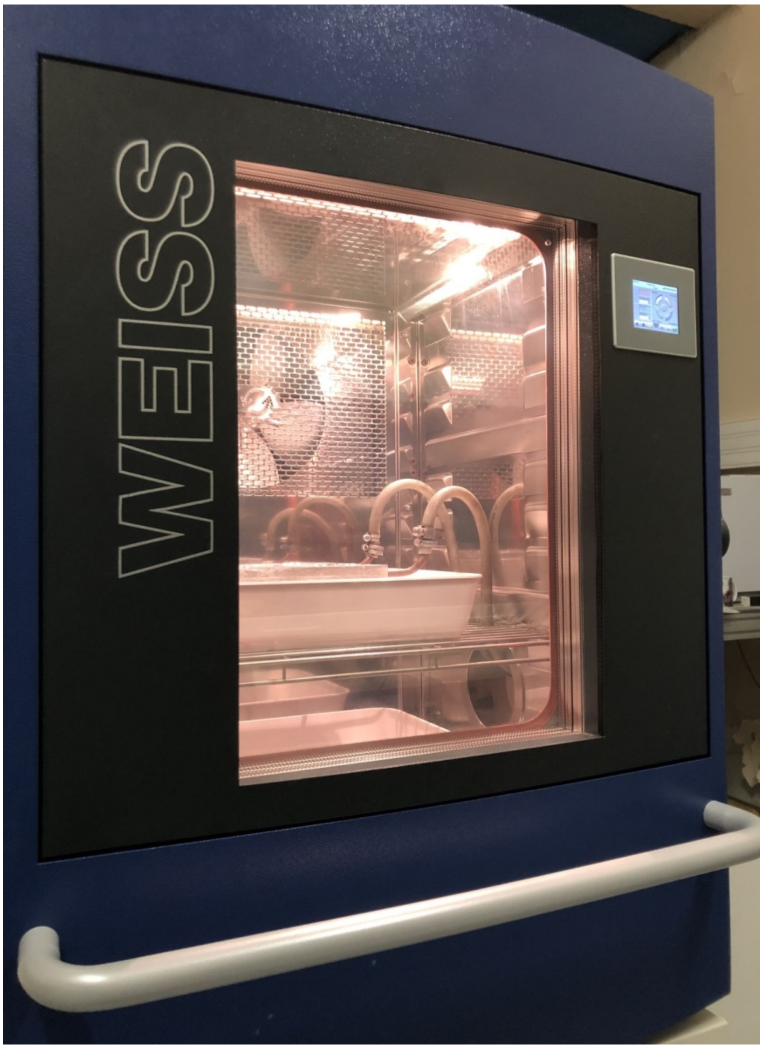

Figure S4. Photo of the experimental instrument for the synthesis of UPA monolith.

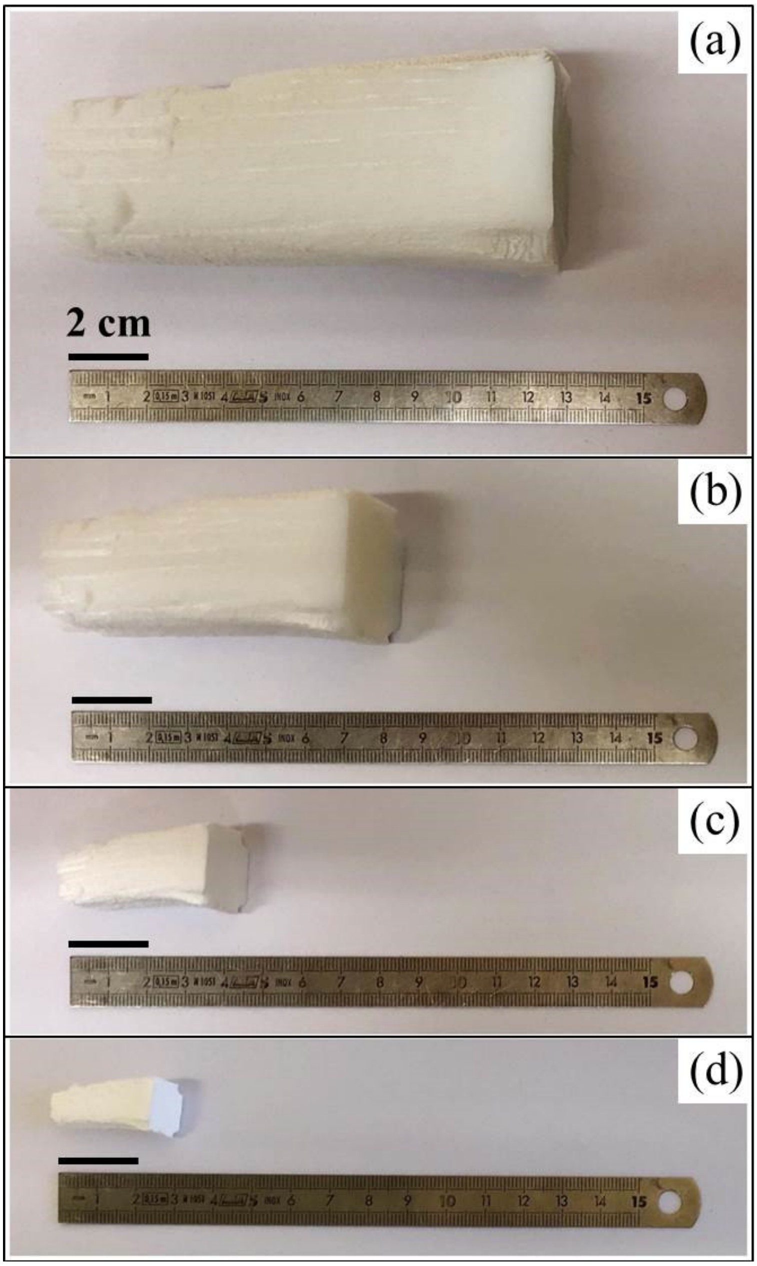

Figure S5. Photos of (**a**) raw fragile UPA, under 4 h of isochronous annealing treatment in air at (**b**) 950 °C, (**c**) 1150 °C, and (**d**) 1350 °C.

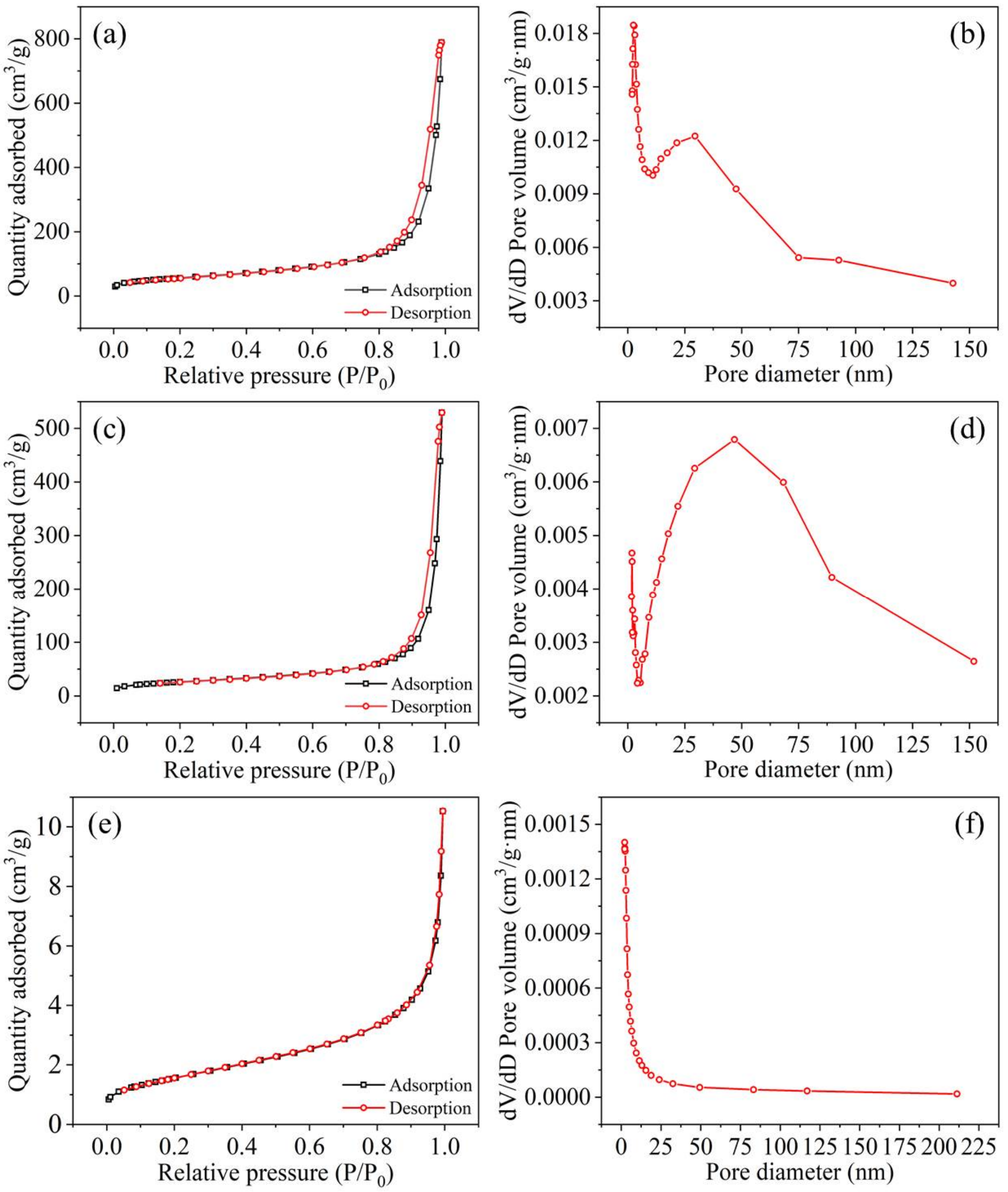

Figure S6. Nitrogen adsorption–desorption isotherms of (**a**) UPA(γ), (**c**) UPA(θ), and (**e**) UPA(*α*) powders. Pore size distributions of (**b**) UPA(γ), (**d**) UPA(θ), and (**f**) UPA(*α*) powders.

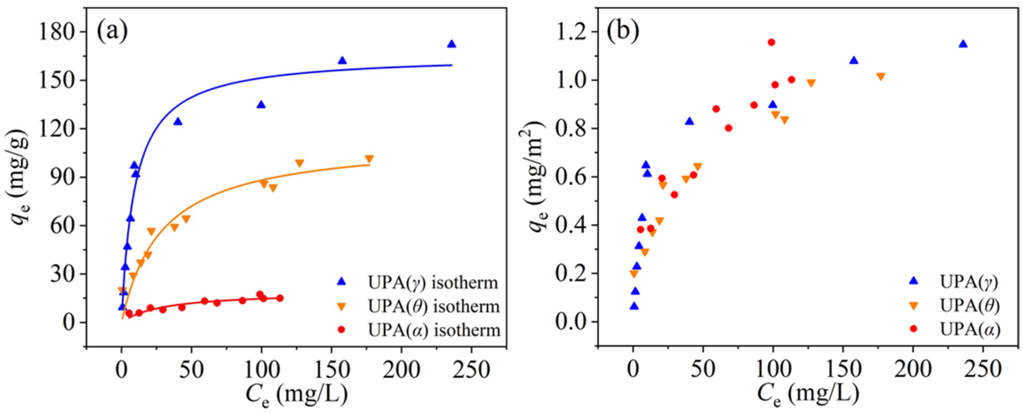

Figure S7. Adsorption isotherm profiles regarding RBBR adsorption capacity with units as (**a**) mg·g⁻¹ and (**b**) mg·m⁻² retained by UPA(γ), UPA(θ), and UPA(*α*) powders. $m/V_{[UPA(\gamma)]} = 5.56$ g·L⁻¹, $m/V_{[UPA(\theta)]} = 5.56$ g·L⁻¹, $m/V_{[UPA(\alpha)]} = 8.33$ g·L⁻¹, initial pH = 4.0 ± 0.1, I = 100 mmol·L⁻¹ sodium acetate, T = 310 K, stirring speed = 150 rpm, and equilibrium time = 24 h.

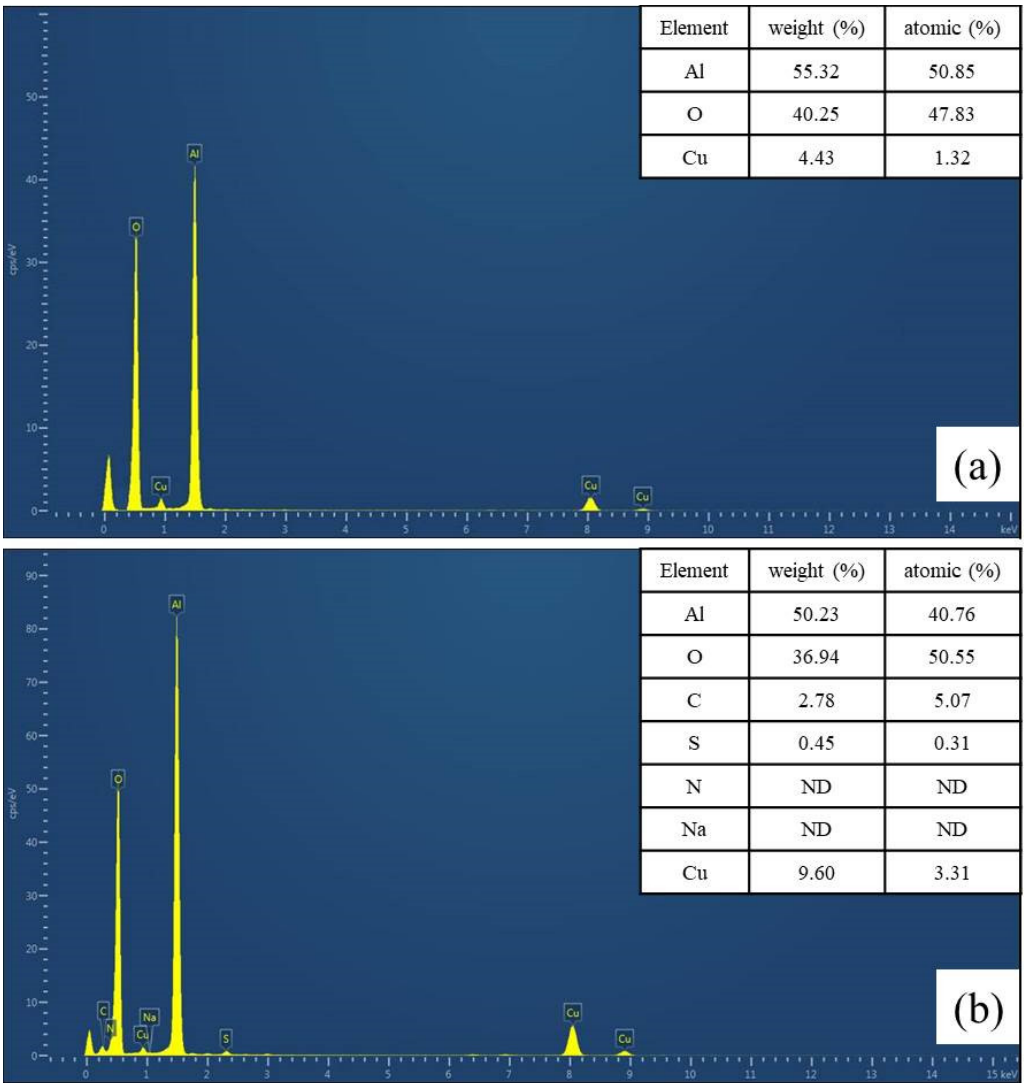

(a)

Element	weight (%)	atomic (%)
Al	55.32	50.85
O	40.25	47.83
Cu	4.43	1.32

(b)

Element	weight (%)	atomic (%)
Al	50.23	40.76
O	36.94	50.55
C	2.78	5.07
S	0.45	0.31
N	ND	ND
Na	ND	ND
Cu	9.60	3.31

Figure S8. EDX spectra of UPA(θ) powders (**a**) before and (**b**) after RBBR adsorption.

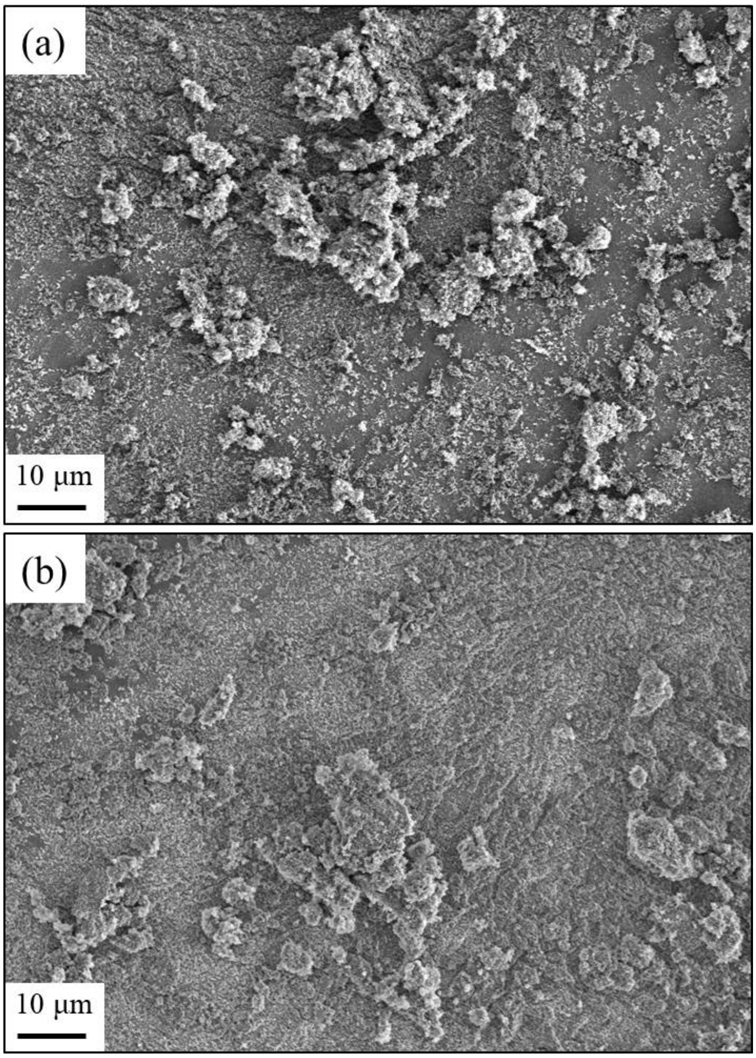

Figure S9. SEM images of UPA(θ) powders (**a**) before and (**b**) after RBBR adsorption.

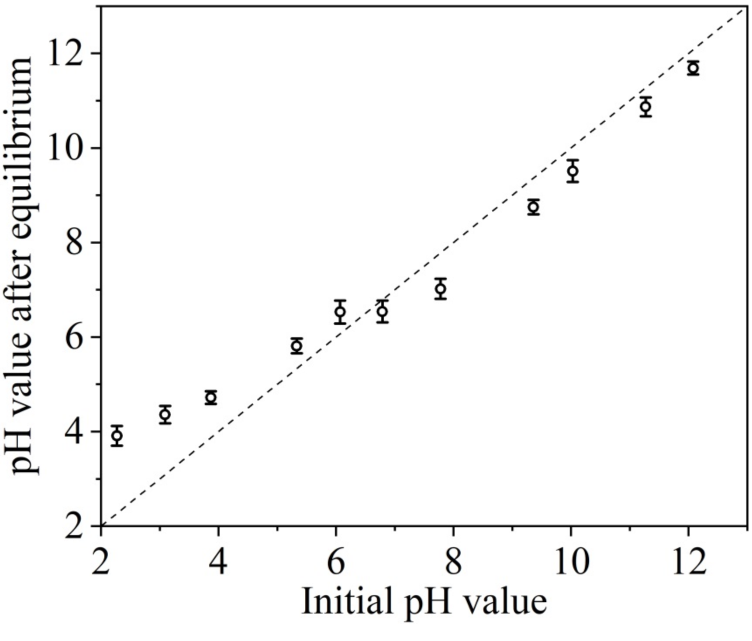

Figure S10. The pH variation after RBBR adsorption equilibrium retained by UPA(θ) powders. $C_{[RBBR]initial}$ = 800 mg·L^{-1}, m/$V_{[UPA(\theta)]}$ = 4.44 g·L^{-1}, I = 100 mmol·L^{-1} sodium acetate, T = 310 K, stirring speed = 150 rpm, and equilibrium time = 24 h.

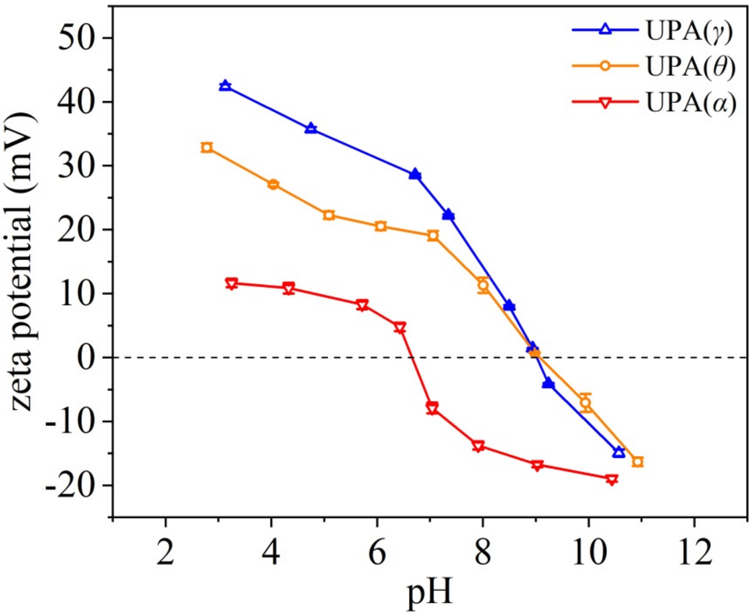

Figure S11. Zeta potential values of UPA(γ), UPA(θ), and UPA(α) powders as a function of pH. $m/V_{[UPA]}$ = 4.44 g·L⁻¹, I = 100 mmol·L⁻¹ sodium acetate.

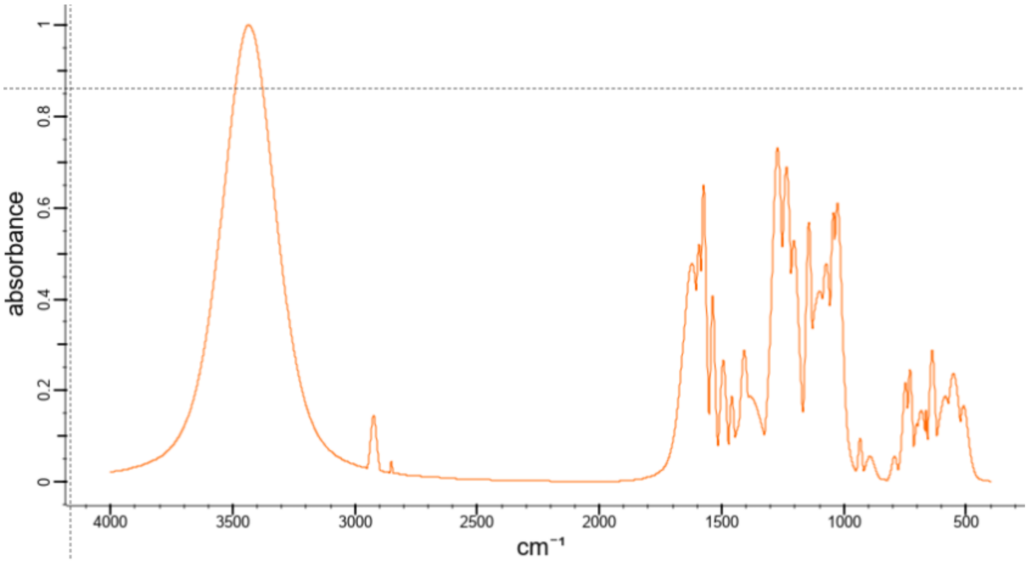

Figure S12. Infrared spectrum of RBBR.

References

1. Vignes, J.-L.; Frappart, C.; Di Costanzo, T.; Rouchaud, J.-C.; Mazerolles, L.; Michel, D. Ultraporous monoliths of alumina prepared at room temperature by aluminium oxidation. *J. Mater. Sci.* **2008**, *43*, 1234–1240.
2. Nguyen, T.H.N. Elaboration and Modifications of Nanofibrous Al_2O_3. Chemical and Process Engineering. Ph.D. Thesis, Université Sorbonne, Paris, France, 2016.
3. Khodan, A.; Nguyen, T.H.N.; Esaulkov, M.; Kiselev, M.R.; Amamra, M.; Vignes, J.-L.; Kanaev, A. Porous monoliths consisting of aluminum oxyhydroxide nanofibrils: 3D structure, chemical composition, and phase transformations in the temperature range 25–1700 °C. *J. Nanopart. Res.* **2018**, *20*, 1–11.
